# Decreased External Skeletal Robustness in Schoolchildren – A Global Trend? Ten Year Comparison of Russian and German Data

**DOI:** 10.1371/journal.pone.0068195

**Published:** 2013-07-23

**Authors:** Katrin Rietsch, Elena Godina, Christiane Scheffler

**Affiliations:** 1 University of Potsdam, Institute of Biochemistry and Biology, Human Biology, Potsdam, Germany; 2 Lomonosov Moscow State University, Institute and Museum of Anthropology, Moscow, Russia; University of Sao Paulo, Brazil

## Abstract

**Objectives:**

Obesity and a reduced physical activity are global developments. Physical activity affects the external skeletal robustness which decreased in German children. It was assumed that the negative trend of decreased external skeletal robustness can be found in other countries. Therefore anthropometric data of Russian and German children from the years 2000 and 2010 were compared.

**Methods:**

Russian (2000/2010 n = 1023/268) and German (2000/2010 n = 2103/1750) children aged 6–10 years were investigated. Height, BMI and external skeletal robustness (Frame-Index) were examined and compared for the years and the countries. Statistical analysis was performed by Mann-Whitney-Test.

**Results:**

Comparison 2010 and 2000: In Russian children BMI was significantly higher; boys were significantly taller and exhibited a decreased Frame-Index (p = .002) in 2010. German boys showed significantly higher BMI in 2010. In both sexes Frame-Index (p = .001) was reduced in 2010. Comparison Russian and German children in 2000: BMI, height and Frame-Index were different between Russian and German children. German children were significantly taller but exhibited a lower Frame-Index (p<.001). Even German girls showed a significantly higher BMI. Comparison Russian and German children in 2010: BMI and Frame-Index were different. Russian children displayed a higher Frame-Index (p<.001) compared with Germans.

**Conclusions:**

In Russian children BMI has increased in recent years. Frame-Index is still higher in Russian children compared with Germans however in Russian boys Frame-Index is reduced. This trend and the physical activity should be observed in the future.

## Introduction

The globalisation leads to intensification of relationships between individuals and countries on an economic, political and cultural base. Changes of lifestyle, food systems and dietary habits are the consequences. The nutrition transition gets to increased energy intake and with the advancement of techniques physical activity decreases. The result is an imbalance of energy intake and consumption which leads to obesity in children and adults in industrial, emergent and developing countries [1].

Worldwide, the physical activity is lower than a few years ago and the reasons for that development are various. Ten year comparisons showed that the physical fitness which is a result of physical activity is reduced in British children and in Czech Republic adolescents [2], [3]. In Germans, only 13.1% of girls and 17.4% of boys were 60 minutes physically active daily [4]. In a country comparison between children from Greece, Netherlands, Belgium, Switzerland and Hungary only 4.6% of girls and 16.8% of boys reached the level of 60 min/day. Swiss children are more physically active than their contemporaries from the other countries [5]. Furthermore, it was represented that Russian children performed better fitness tests than Americans. Russian children spent more time in structured training sports clubs and walk to and from school [6]. Probably, a development which has been enforced from the end of the 90^th^ where only 33% of the Russian households held a car and no school busses were available, so 92% of the children went to school by walking [7]. In other countries as US, Canada, UK and Australia active commuting decreased in the last years which, however, might be a factor to raise daily physical activity [8], [9], [10]. Even media consumption as a consequence thereof sedentary behaviour is increasing [11]. Further, children in Eastern European states with a low social-economic status also spent more time with TV viewing and they participated in the sports club less frequently with the result of lower physical activity [12], [13].

Reduced physical activity is not only one of the reasons for obesity but also affects external skeletal robustness which is decreased [14], [15]. A ten year comparison showed that new trend in German 6–12 years-olds boys and girls [16]. However, physical activity is needed to boost the bone growth beside calcium intake especially at an early age [17], [18], [19]. Children with a decreased external skeletal robustness will have it their whole lifetime. When they are seniors and especially overweight the prevalence of joint diseases and osteoporosis may increase. This would result in a high cost factor for the health system.

In that context questions arise whether that negative trend of decreased external skeletal robustness is a global development such as obesity and how the trend proceeds as compared to other countries. In this study anthropometric data of German children were compared with data of Russian children from 2000 and 2010. Due to the globalisation, the political development, the lower physical activity in Eastern Europe states and the fact that Russian children are fitter than the American children we suggest that the negative changes reach the Russian population after a time delay. We assumed differences between Russian and German data in 2000 and an approach in 2010.

## Materials and Methods

### Ethics statement

Investigations were approved by relevant institutions: Senate Department of Education, Science and Research Berlin (Permit number: VI D 1), Ministry of Education, Youth and Sports Brandenburg (Permit number: 60/2010) and Department of Education of Moscow city. The study was implemented on a voluntary basis with parents' permission. They signed a written informed consent but finally the children should agree with participation as well. All data was anonymized.

### Samples

In Russia and Germany children aged from 6 to 10 completed years were anthropometric examined in 2000 and 2010. Allocation of samples in sex and nationality is represented in Table 1. Measurements were taken at elementary schools in Moscow (Russia), Brandenburg and Berlin (Germany). Schools were from different districts therefore children were from varying social backgrounds.

**Table 1:** Sample allocation of Russian and German children 2000/2010.

		age					
year/country/sex		6	7	8	9	10	Σ
**2000**	Russian all	69	267	279	276	132	1023
	Russian girls	38	117	137	118	68	478
	Russian boys	31	150	142	158	64	545
	German all	407	333	386	487	490	2103
	German girls	177	175	209	248	241	1050
	German boys	230	158	177	239	249	1053
**2010**	Russian all	3	93	68	59	45	268
	Russian girls	0	44	33	21	18	116
	Russian boys	3	49	35	38	27	152
	German all	226	407	358	426	333	1750
	German girls	121	204	201	211	163	900
	German boys	105	203	157	215	170	850

### Anthropometric measurements

Anthropometric measurements were followed by standardized methods in a standing position with prescribed measuring instruments [20]. The anthropometric parameters height, weight and elbow breadth were taken. By means of this, the following indices were calculated and compared:

1. Body Mass Index 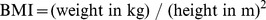

2. External skeletal robustness [21]

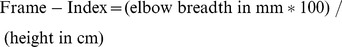
Through these Index it can be concluded on external skeletal robustness. Three types small, medium and large frame size can be realized by creating percentile curves. However, to analyse Frame-Index 3^rd^, 10^th^, 50^th^, 90^th^ and 97^th^ percentiles were calculated and the curves were smoothed with LMS method [22]. We used these percentiles due to the 3^rd^ and 10^th^ percentiles showed significant differences in German children in the years 2000 and 2010 [16]. Although sample size was small in Russian children in 2010 these percentiles were applied for the comparison.

### Statistical analysis

Samples sizes of the different years 2000/2010 and States Russian/German differ greatly (Table 1). Data was partly not normally distributed which showed the Kolmogorow-Smirnow Test. Therefore to determine differences between the groups non-parametric test (Mann-Whitney-Test) was used. The following significance levels were used: p<.001 (***), p<.01 (**), p<.05 (*). Statistical analysis was realized by the program SPSS 19 IBM.

## Results

### Russian children: Comparison 2000 and 2010

**Figure 1:**
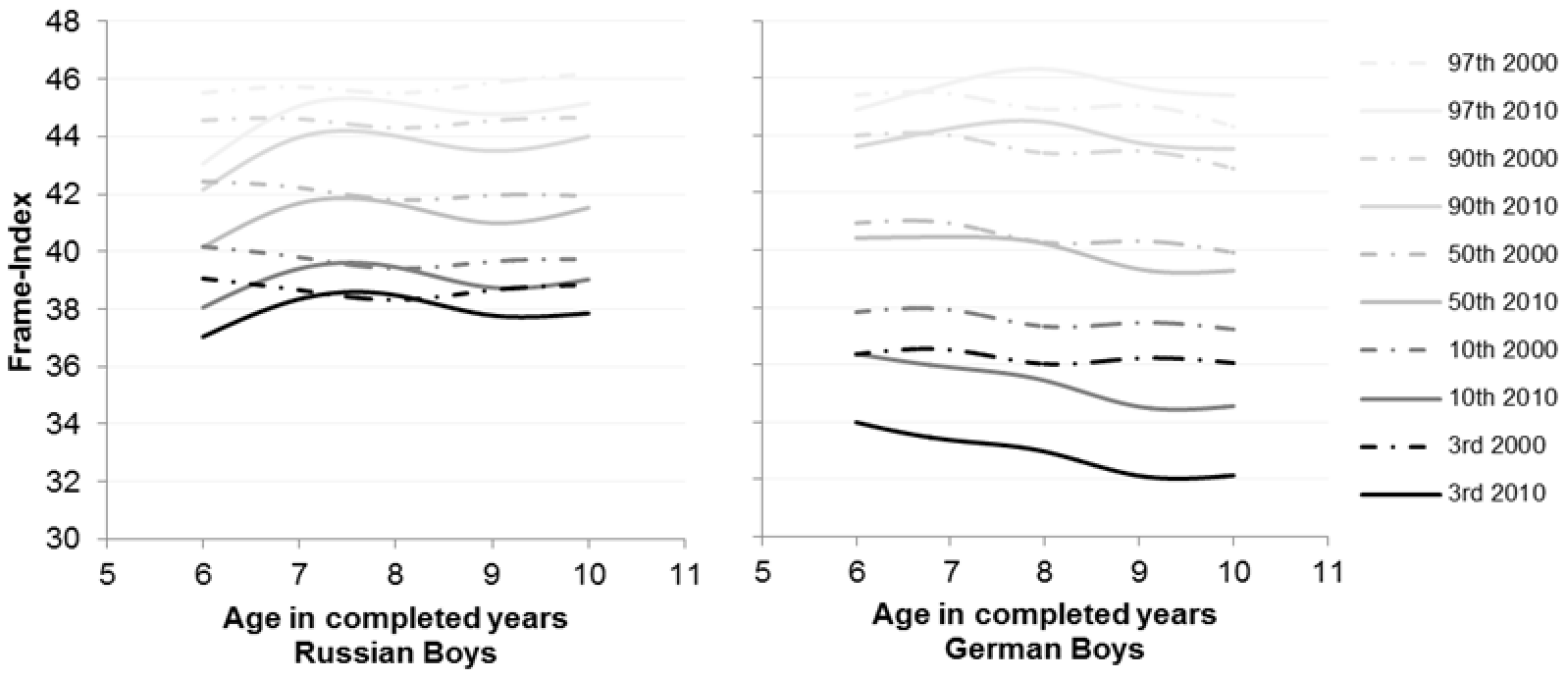
Percentiles of the parameter Frame-Index for Russian and German boys for the years 2000 (dashed lines) and 2010 (solid lines).

In Russian children the comparison between 2000 and 2010 showed that only BMI (U = 109163, p<.001) was distinguished but not height (U = 130448, p = .222) and Frame-Index (U = 102801, p = .343). The results were reflected in girls, only BMI (p<.001) was significantly higher in 2010 (BMI: p50 = 16.79 m^2^/kg) than in 2000 (BMI: p50 = 15.99 m^2^/kg) (Tables 2, 3). Russian girls were taller till the age of 10 in 2000. Frame-Index did not differ between the years. However, Russian boys were taller (height: p50 = 134.7 cm; p = .034) and exhibited a higher BMI (p<.001) in almost every age group in 2010. Frame-Index was decreased (2000: p50 = 42.1 vs. 2010: p50 = 41.41; p = .002) (Table 2, Fig. 1).

**Table 2:** P50 and mean values ± standard deviation of the parameters BMI (kg/m^2^), Frame-Index, height (cm) of Russian boys and girls from the years 2000 and 2010.

		Russian boys				Russian girls			
		2000		2010		2000		2010	
age	parameter	p50	Mean ± SD	p50	Mean ± SD	p50	Mean ± SD	p50	Mean ± SD
**all**	**BMI**	16.21	16.67±2.16	16.82	17.48±2.57	15.99	16.34±2.15	16.79	17.29±2.65
	**Frame-Index**	42.1	42.06±1.91	41.41	41.48±1.87	40.17	40.22±2.03	40.15	40.51±2.14
	**height**	132.7	132.17±8.06	134.7	134.2±8.27	132.1	131.86±7.69	131.5	132.05±8.57
**6**	**BMI**	15.82	15.93±1.52	/	/	15.19	15.89±1.92	/	/
	**Frame-Index**	42.59	42.43±1.56	/	/	41.14	41.43±1.74	/	/
	**height**	122.9	122.41±4.11	/	/	121.6	122.87±4.59	/	/
**7**	**BMI**	15.7	16.22±2.05	16.33	16.72±2.1	15.89	16.02±1.96	17.06	17.18±2.53
	**Frame-Index**	42.29	42.2±1.97	41.5	41.68±1.93	40.38	40.42±2.01	40.61	40.89±2.06
	**height**	126.2	125.59±5.36	127.7	128.28±5.67	126.2	126.72±5.4	125.2	126.14±5.76
**8**	**BMI**	16.38	16.05±1.97	16.52	17.22±2.44	16.11	16.3±2.14	16.78	17.19±2.37
	**Frame-Index**	41.82	41.83±1.90	41.63	41.69±1.58	39.82	40±2.13	39.74	40.34±2.44
	**height**	132.9	132.18±5.89	131.8	131.74±6.8	132.6	131.81±5.91	130.5	130.62±5.56
**9**	**BMI**	16.56	17.01±2.22	17.86	18.24±3.18	16.25	16.07±2.38	16.67	16.72±2.24
	**Frame-Index**	41.9	42.04±1.9	40.86	41.09±2.02	40.18	39.95±2.01	40.15	40.29±2.07
	**height**	137.1	136.75±5.64	140.4	140.54±6.34	137.5	136.32±6.39	135.4	137.26±6.95
**10**	**BMI**	16.93	17.79±2.51	18.1	18.15±2.39	15.98	16.32±2.19	17.37	18.45±3.63
	**Frame-Index**	41.9	42.09±1.99	41.72	41.52±1.94	39.88	40.03±1.70	39.75	40.11±1.79
	**height**	142.2	142.01±5.56	139.4	140.51±5.93	138.8	139.65±5.2	142.6	143.06±6.46

**Table 3:** Data comparison of the parameters BMI (kg/m^2^), Frame-Index, height (cm) of the years 2000 and 2010 for Russian and German boys and girls children.

		2000 vs. 2010							
		Russian boys		Russian girls		German boys		German girls	
age	parameter	U	p	U	p	U	p	U	p
**all**	**BMI**	33535	<.001***	21732	<.001***	410562	.002**	464152	.528
	**Frame-Index**	26055	.002**	20883	.290	406989	.001**	433953	.002**
	**height**	36769	.034*	26683	.530	436508	.355	430634	.001**
**6**	**BMI**	n.d.a	n.d.a	n.d.a	n.d.a	10556	.065	10260	.621
	**Frame-Index**	n.d.a	n.d.a	n.d.a	n.d.a	10810	.139	8912	.014*
	**height**	n.d.a	n.d.a	n.d.a	n.d.a	11537	.513	10668	.956
**7**	**BMI**	2830	.016*	1941	.016*	15981	.955	17115	.490
	**Frame-Index**	2251	.115	1929	.241	14228	.066	17124	.495
	**height**	2883	.024*	2417	.553	15110	.346	16894	.369
**8**	**BMI**	2199	.292	1764	.050	11903	.024*	19082	.109
	**Frame-Index**	1976	.756	1572	.519	13685	.812	20215	.564
	**height**	2244	.376	1891	.146	12969	.293	16748	<.001***
**9**	**BMI**	2301	.026*	1190	.819	23423	.104	24263	.180
	**Frame-Index**	1541	.009**	958	.574	21648	.004**	23701	.082
	**height**	1949	.001**	1216	.892	24386	.349	24640	.282
**10**	**BMI**	708	.175	419	.048*	19817	.268	16885	.017*
	**Frame-Index**	553	.363	420	.766	19028	.079	17776	.105
	**height**	679	.109	430	.064	18802	.052	17802	.110

U = Mann-Whitney-Test.

p = p-value. significant in bold.

significance levels = p<.001 (***), p<.01 (**), p<.05 (*).

n.d.a = no data available for Russian children.

### German children: Comparison 2000 and 2010

**Figure 2:**
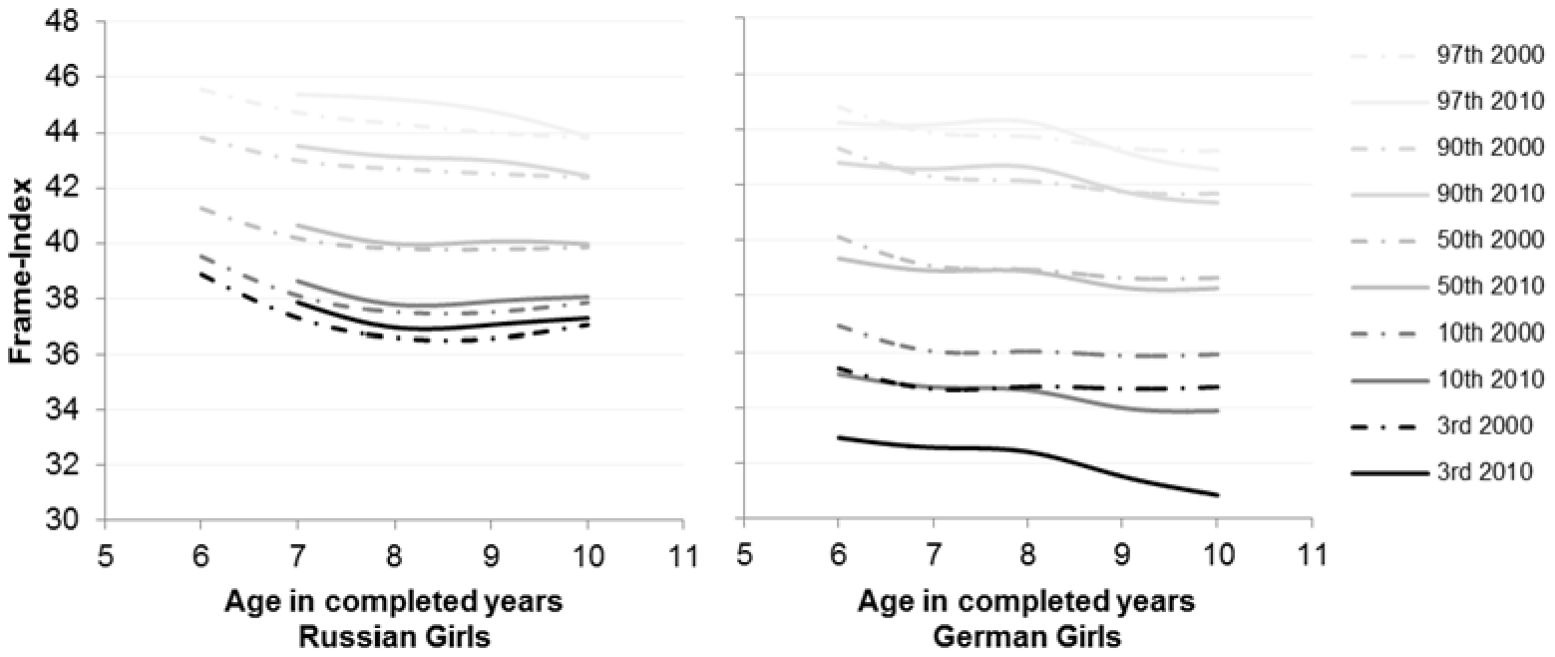
Percentiles of the parameter Frame-Index for Russian and German girls for the years 2000 (dashed lines) and 2010 (solid lines).

In German children height (U = 1734657, p = .002), Frame-Index (U = 1668006, p<.001) and BMI (U = 1749894, p = .009) were significantly different in 2000 and 2010. German boys showed a higher BMI (p = .002) but a decreased Frame-Index (p = .001) especially at the 3^rd^ and 10^th^ (Fig. 1) percentiles in 2010. Height did not differ between the years in contrast to the German girls (2000: p50 = 135 cm vs. 2010: p50 = 132.7 cm; p = .001). In girls, BMI did not vary over the years (p = .528) but Frame-Index decreased like in boys and the same centiles (Tables 3, 4, Fig. 2).

**Table 4:** P50 and mean values ± standard deviation of the parameters BMI (kg/m^2^), Frame-Index, height (cm) of German boys and girls from the years 2000 and 2010.

		German boys				German girls			
		2000		2010		2000		2010	
		p50	Mean ± SD	p50	Mean ± SD	p50	Mean ± SD	p50	Mean ± SD
**all**	**BMI**	16.21	16.85±2.56	16.52	17.1±2.59	16.47	16.97±2.53	16.49	17.04±2.59
	**Frame-Index**	40.38	40.49±2.36	40.24	39.71±3.49	38.95	39.09±2.42	38.86	38.46±3.16
	**height**	134.5	134.68±10.46	133.9	134.22±9.42	135	134.77±10.36	132.7	133.25±9.66
**6**	**BMI**	15.4	15.77±1.86	15.82	16.02±1.94	15.75	15.93±1.82	15.61	15.89±2.04
	**Frame-Index**	40.84	40.92±2.45	40.56	40.28±2.81	40.3	40.12±2.45	39.69	39.13±3.04
	**height**	123	123.02±5.49	123.8	123.7±5.79	122	121.47±5.53	121.2	121.5±5.68
**7**	**BMI**	15.87	16.29±1.98	15.81	16.21±2.17	15.85	16.33±2.47	15.93	16.39±2.21
	**Frame-Index**	40.75	40.96±2.23	40.61	40.11±3.47	39.1	39.12±2.55	39.1	38.75±3.06
	**height**	127.7	128±6.3	127	127.3±5.43	127	127.4±5.97	126.4	126.85±5.42
**8**	**BMI**	16.02	16.65±2.25	16.39	17.08±2.24	16.94	17.15±2.24	16.39	16.92±2.38
	**Frame-Index**	40.46	40.31±2.41	40.63	40.11±3.28	39.16	39.03±2.39	39.01	38.69±3.24
	**height**	134	134.1±6.13	133.2	133.15±6.14	134.2	134.33±6.32	131.8	132.19±6.05
**9**	**BMI**	16.6	17.4±2.77	16.88	17.76±2.79	16.73	17.3±2.77	17.03	17.49±2.84
	**Frame-Index**	40.37	40.41±2.45	39.94	39.18±3.89	38.47	38.73±2.26	38.4	38.01±3.15
	**height**	139.5	140±7.03	138.8	139.33±7.03	139.65	139.73±6.58	139.1	139.1±6.58
**10**	**BMI**	17.09	17.83±2.92	17.49	18.01±2.86	17.11	17.69±2.66	17.78	18.28±2.71
	**Frame-Index**	39.82	39.98±2.14	39.57	39.17±3.4	38.65	38.74±2.28	38.59	37.87±3.16
	**height**	144.5	144.93±7.02	144.1	143.47±6.72	144.5	144.75±6.78	143.1	143.68±6.83

### Russian and German children in 2000

In 2000 between Russian and German children, BMI (U = 998556, p = .001), Frame-Index (U = 565949, p<.001) and height (U = 929587, p<.001) were significantly different. This result can be found in girls as well. German girls were taller (German: p50 = 135 cm vs. Russian: p50 = 132.1 cm), exhibited a higher BMI (German: p50 = 16.47 m^2^/kg vs. Russian: p50 = 15.99 m^2^/kg) but a lower Frame-Index (German: p50 = 38.95 vs. Russian: p50 = 40.17). In every age group a significant difference in Frame-Index can be found (Tables 2, 4, 5, Fig. 2). This was also shown in boys (Tables 2, 4, Fig. 1). Russian boys had a higher Frame-Index (German: p50 = 40.38 vs. Russian: p50 = 42.1) but were smaller than German boys (German: p50 = 134.5 cm vs. Russian: p50 = 132.7 cm) in 2000. In 2010, BMI (U = 216372, p = .043) and Frame-Index (U = 149073, p<.001) were distinguished but not height (U = 227552, p = .434).

**Table 5:** Data comparison of the parameters BMI (kg/m^2^), Frame-Index, height (cm) of Russian and German boys and girls per year 2000 and 2010.

		Russian vs. German							
		2000 boys		2000 girls		2010 boys		2010 girls	
age	parameter	U	p	U	p	U	p	U	p
**all**	**BMI**	282725	.630	217947	<.001***	59011	.089	49013	.292
	**Frame-Index**	129504	<.001***	146007	<.001***	43494	<.001***	32166	<.001***
	**height**	252018	<.001***	213033	<.001***	64407	.953	47849	.144
**6**	**BMI**	3376	.632	3180	.599	n.d.a	n.d.a	n.d.a	n.d.a
	**Frame-Index**	1873	.001**	2022	.005**	n.d.a	n.d.a	n.d.a	n.d.a
	**height**	3125	.265	2923	.207	n.d.a	n.d.a	n.d.a	n.d.a
**7**	**BMI**	10963	.256	10153	.905	4282	.131	3546	.029*
	**Frame-Index**	5786	<.001***	5944	<.001***	3603	.003**	2656	<.001***
	**height**	9769	.008**	9221	.151	4473	.275	3942	.206
**8**	**BMI**	12138	.600	11274	.001**	2700	.873	3019	.409
	**Frame-Index**	6226	<.001***	8410	.002**	1818	.002**	2389	.010*
	**height**	10976	.052	11704	.004**	2347	.178	2842	.188
**9**	**BMI**	17963	.412	12562	.039*	3714	.373	1809	.166
	**Frame-Index**	7601	<.001***	8470	<.001***	2845	.003**	1245	.001**
	**height**	13906	<.001***	11143	<.001***	3592	.236	1814	.171
**10**	**BMI**	7745	.730	6150	.002**	2141	.576	1395	.733
	**Frame-Index**	2788	<.001***	3966	<.001***	1254	<.001***	826	.002**
	**height**	5875	.001**	4566	<.001***	1590	.010*	1405	.771

U = Mann-Whitney-Test.

p = p-value. significant in bold.

significance levels = p<.001 (***), p<.01 (**), p<.05 (*).

n.d.a = no data available for Russian children.

### Russian and German children in 2010

In contrast to the 2000 analysis in 2010 in children of both sexes only Frame-Index was significantly different in every age group (Table 5). Russian boys (German: p50 = 40.24 vs. Russian: p50 = 41.41) and girls (German: p50 = 38.86 vs. Russian: p50 = 40.15) had a higher Frame-Index than the Germans (Tables 2, 4, Fig. 1, 2).

## Discussion

It is established that the inclination of obesity and especially the increased body fat deposition is a result of genetic factors [23], [24]. Otherwise environmental factors as high-calorie nutrition, physical activity and sedentary behaviour affect body fat production as well. An imbalance of these components leads to overweight. This development can be found in different population and has been evolved into a global problem [1]. Apart from this trend another new development can be shown in relation to the skeleton of the German children. The external skeletal robustness has decreased. Each element of the skeletal system as bone mass and density will be influenced by genetic factors. Furthermore environmental factors as calcium intake and physical activity affect on them [25], [26], [19]. This is likewise to the body fat deposition. Now we displayed a trend concerning to the skeletal system. Between two different populations (German/Russian) and within the population external skeletal robustness, BMI and height were compared. It was supposed that differences were existed between Russian and German data in 2000 and an approach in 2010. In 2010, BMI and height of the Russian children were adapted on the values of the German children while in 2000 differences existed. In Russian children BMI were increased due to the changed nutrition. More than a half of the calories were ingested in form of bread, pastries, sugar and potatoes [27]. Furthermore, the secular trend can be observed in Russian children especially in boys. Physical height increased in Russian children due to advance of socio-economic conditions [28]. Russian girls aged 10 were 3.5 cm higher on average in 2010 than in 2000. Unpublished data showed that sexual maturity began at the same age in 2010. In contrast German children were a little shorter in 2010 than in 2000. In German girls the differences between each age group vary in 2010 and 2000. This might be a sampling problem. Nevertheless, Scheffler (2011) showed the same results of decreased height. One explanation is that the environmental conditions are optimal and the genetic potential of body height has been achieved in industrialized countries [16]. In 2000 as well in 2010, Russian children exhibited a higher external skeletal robustness as compared with German children. This finding can be arising from genetic factors but also due to the dosage of physical activity. We assumed that physical activity is higher in Russian children than in German children. However, at present no data is available to consider that assumption. Though, Hastie et al. [6] exhibited that Russian children were fitter than their contemporaries in the US. After all, in 2010 compared with 2000 in Russian boys' external skeletal robustness were decreased whereas that negative development can be found in both sexes of the German children. Although the Russian children may be more physically active than the German children the development of a reduced physical activity may exist. In case of reduced physical activity in Russian children it affects boys at first. Environmental factors impact boys' body composition stronger than girls [29]. Also according to one study Moscow girls are more physically active than boys [30].

## Conclusions

In Russian boys both negative developments the increasing prevalence of obesity and the reduction of external skeletal robustness can be observed. In this context physical activity should be particularly investigated.
